# Extended interval dosing strategies in multiple sclerosis: insights from natalizumab and ocrelizumab trials

**DOI:** 10.1007/s00415-024-12273-9

**Published:** 2024-03-04

**Authors:** Yasmin Zid, Neil P. Robertson

**Affiliations:** 1 Department of Neurology, University Hospital of Wales, Heath Park, Cardiff, CF14 4XN UK; 2 Institute of Psychological Medicine and Clinical Neuroscience, Cardiff University, University Hospital of Wales, Heath Park, Cardiff, CF14 4XN UK

Recent advances in the treatment of relapsing multiple sclerosis (RRMS) have seen the emergence of a wide range of high efficacy disease-modifying therapies (DMTs) including natalizumab and ocrelizumab, which are now well embedded in routine clinical practice. However, despite their undoubted efficacy in reducing disease activity and relapse rates, challenges persist regarding dosing regimens and potential adverse effects. Natalizumab, administered intravenously every 4 weeks, carries a small but significant risk of progressive multifocal leukoencephalopathy (PML) with risk factors including prolonged dosing, a high JC index or previous chemotherapy. Ocrelizumab, administered every 6 months, has raised long-term concerns concerning B cell repopulation dynamics, acquired immunodeficiency, and vaccine efficacy. It is thought that some of these risks could be modified by adaptation of existing dosing regimens without sacrificing efficacy and have become the focus of recent clinical trials. This month's journal club explores some of the recent studies in this field and discusses implications for clinical practice.

The first paper compares the effects of extended interval dosing (EID), with standard interval dosing (SID) on brain atrophy. The second paper addresses similar questions about ocrelizumab and its effects on B-cell repopulation. The third paper, the NOVA trial, assesses the safety and efficacy of switching natalizumab dosing frequency from once every four weeks to once every six weeks in patients with relapsing multiple sclerosis (RRMS). The final paper from the NEXT-MS trial provides insights into tailoring treatment approaches based on individual patient responses, thereby further enhancing treatment outcomes.

## Extended interval dosing of ocrelizumab modifies the repopulation of B cells without altering the clinical efficacy in multiple sclerosis

This study explored the effects of ocrelizumab, a monoclonal antibody targeting CD20-positive B cells, on the immune system of MS patients. Using CyTOF technology, researchers extensively analysed immune cell subsets in patients undergoing ocrelizumab treatment, focusing on B cell repopulation dynamics and the impact of standard vs. extended dosing intervals. The study was a prospective observational cohort study. Patients were recruited from a single centre in Holland. A total of 108 patients with MS were included in the study. Among them, 28 constituted the control group (CG), 43 were under SID of ocrelizumab, and 37 were under EID of ocrelizumab. The inclusion criteria involved patients receiving personalized dosing of ocrelizumab based on CD19+ B cell counts measured via flow cytometry. Patients in the CG were on other disease-modifying therapies (DMTs) but planned to switch to ocrelizumab.

Blood samples were drawn every 4 weeks, starting 24–30 weeks after the administration of ocrelizumab to monitor CD19+ B cell counts and decide on timing of subsequent doses. Several clinical parameters were assessed, including expanded disability status scale (EDSS), relapse rates, brain magnetic resonance imaging (MRI), and serum neurofilament light (sNfL) levels. Demographic and clinical characteristics of the participants were recorded and adjustments for covariates such as age, sex, and type of MS made, and p-values adjusted for multiple comparisons. Significant differences were considered at p < 0.05.

Key findings were that both standard and extended dosing intervals were effective in treating MS, with no significant correlation between dosing intervals and disease outcomes. B cell repopulation after ocrelizumab treatment primarily comprised immature and transitional B cells, with slightly different dynamics observed between standard and extended dosing intervals. Patients on extended dosing intervals exhibited a trend of faster B cell repopulation within two weeks post-treatment compared to those on standard intervals. Furthermore, repopulated B cells, particularly after extended dosing intervals, showed increased expression of migratory markers, suggesting a heightened migratory capacity towards the CNS. Additionally, higher CD20 expression correlated with more effective B cell depletion in consecutive extended dosages, hinting at potential strategies to enhance therapeutic efficacy.

### Comment

This study supports effectiveness of both standard and extended dosing regimens. The authors suggest extending dosing intervals as a potential strategy to minimise B-cell repopulation without compromising efficacy. However, the authors acknowledge limitations such as a heterogeneous control group and lack of longitudinal samples. The study also sheds some light on ocrelizumab pharmacodynamics, including B-cell repopulation, migratory behaviour, and implications of dosing intervals. It advocates for personalised dosing strategies tailored to individual patient responses and emphasises the need for further exploration to optimise treatment outcomes.

Rodriguez-Mogeda C, et al. J Neuroinflammation. 2023 Sep 26;20(1):215. doi: https://doi.org/10.1186/s12974-023-02900-z. PMID: 37752582; PMCID: PMC10521424.

## Exploring the effects of extended interval dosing of natalizumab and drug concentrations on brain atrophy in multiple sclerosis

This paper explores the impact of natalizumab EID on brain atrophy. Despite natalizumab's effectiveness in suppressing MS disease activity, disability progression and neurodegeneration can still occur during treatment.This study is a retrospective single centre cohort study, aimed at exploring the longitudinal effects of EID of natalizumab compared to SID on brain atrophy in patients with relapsing MS.

The study included 82 RRMS patients fulfilling 2017 McDonald criteria. Fifty were on natalizumab SID and 32 on EID. Inclusion criteria included at least two available MRI scans with minimum follow-up of 2 years, and availability of serum samples. Primary endpoints were change in whole brain, ventricular, and thalamic volume over time measured via 3D-FLAIR-MRI scans. Over a median follow-up of 3.2 years, no significant differences in annualised volume change between EID and SID groups for the whole brain (− 0.21% vs − 0.16%, p = 0.42), ventricular (1.84% vs 1.13%, p = 0.24), and thalamic (− 0.32% vs − 0.32%, p = 0.97) were observed.

### Comment

The authors conclude that natalizumab EID can effectively reduce treatment burden and associated risks without compromising its efficacy in controlling MS disease activity. Limitations of the study include its retrospective design, small sample size, and short follow-up duration. Additionally, the heterogeneity in MRI scanners and acquisition protocols may have introduced biases in assessing brain volume measures, although efforts were made to correct for these factors. Further research in larger cohorts with longer follow-up periods is warranted to confirm these findings.

Toorop AA, et al., Mult Scler. 2024 Feb;30(2):266-271. doi: https://doi.org/10.1177/13524585231225855. Epub 2024 Jan 18. PMID: 38235514; PMCID: PMC10851624

## Comparison of switching to 6-week dosing of natalizumab versus continuing with 4-week dosing in patients with relapsing-remitting multiple sclerosis (NOVA): a randomised, controlled, open-label, phase 3b trial

NOVA is a phase 3b, randomized, controlled, open-label, rater-blinded trial conducted across 89 MS centres in 11 countries spanning the Americas, Europe, and Western Pacific. The study aimed to evaluate the safety and efficacy of natalizumab dosing once every 6 weeks compared to the standard dosing regimen of once every 4 weeks in patients with RRMS.

A total of 499 participants were enrolled between December 26th 2018, and August 30th 2019, out of 605 assessed for eligibility. Inclusion criteria were; aged 18–60 years, diagnosed with RRMS, and had received intravenous natalizumab 300 mg once every 4 weeks with no relapses for at least 12 months prior to randomization, with no missed doses in the previous 3 months.

The primary endpoint of the study was the number of new or newly enlarging T2 hyperintense lesions at week 72, assessed in participants who received at least one dose of assigned treatment and had at least one postbaseline MRI, relapse, or neurological examination or efficacy assessment. The study used two prespecified estimands to handle missing data: the primary estimand included all data regardless of treatment continuation, while the secondary estimand considered data after treatment discontinuation or study withdrawal as missing. Safety was assessed in all participants who received at least one dose of study treatment.

The results indicated that the mean number of new or newly enlarging T2 hyperintense lesions at week 72 was numerically higher in the once every 6 weeks group compared to the once every 4 weeks group, reaching statistical significance under the secondary estimand (p = 0.044). However, the primary estimand did not show a statistically significant difference (p = 0.076). Adverse events occurred in 78% of participants in the once every 6 weeks group and 77% in the once every 4 weeks group, with similar rates of serious adverse events and no reported deaths. One case of asymptomatic progressive multifocal leukoencephalopathy was reported in the once every 6 weeks group.

Comment: the NOVA trial demonstrated a numerical difference in lesion outcomes between the two dosing regimens, with safety profiles being similar. The authors suggest that most patients stable on natalizumab once every 4 weeks can switch to once every 6 weeks without clinically meaningful loss of efficacy, although clearly vigilance in monitoring for adverse events, including progressive multifocal leukoencephalopathy, remains crucial.

Foley JF, et al. Lancet Neurol. 2022 Jul;21(7):608-619. doi: https://doi.org/10.1016/S1474-4422(22)00143-0. Epub 2022 Apr 25. PMID: 35483387.

## Prospective trial of natalizumab personalised extended interval dosing by therapeutic drug monitoring in relapsing-remitting multiple sclerosis (NEXT-MS)

NEXT-MS trial is a prospective phase IV non-randomized study, aiming to assess the efficacy and safety of individualized EID of natalizumab in individuals diagnosed with RRMS. A total of 376 adults who had received at least 6 natalizumab infusions were enrolled across 21 sites in the Netherlands. Participants were divided into three groups: personalized EID targeting a drug trough concentration of 10 µg/mL (EID10), an exploratory group with a target of 5 µg/mL (EID5), and a (SID) group receiving infusions every 4 weeks.

The primary outcome measure was radiological disease activity, specifically the occurrence of new or newly enlarged T2 lesions. Comparison was primarily between the EID10 group and a historical control group receiving standard dosing every 4 weeks (HSID). Additionally, secondary endpoints included physical disability assessed by EDSS, relapse frequency, serum neurofilament light chain concentrations, and the development of seropositivity for JC virus.

Results from the initial phase of the trial demonstrated that the incidence rate of radiological disease activity in the EID10 group was 10.0 per 1000 person-years, which was deemed non-inferior to the HSID group (24.7 per 1000 person-years). The EID5 group showed an incidence rate of 10.0 per 1000 person-years, while the SID group had an incidence rate of 47.0 per 1000 person-years. Notably, serum neurofilament light levels did not increase over time within the EID groups, and there were no reported cases of progressive multifocal leukoencephalopathy.

### Comment

No cases of progressive multifocal leukoencephalopathy were reported in any group. These findings suggest that personalized EID of natalizumab can effectively control MS disease activity while potentially allowing for longer treatment intervals. The study provides insights into optimizing natalizumab dosing strategies, with implications for improving treatment outcomes and patient management in MS.

Toorop AA, et al. J Neurol Neurosurg Psychiatry. 2023 Nov 14:jnnp-2023-332119. doi: https://doi.org/10.1136/jnnp-2023-332119. Epub ahead of print. PMID: 37963723.

### Summary

Extended interval dosing (EID) strategies for high-efficacy disease-modifying therapies represent a promising avenue in MS. Recent studies focusing on natalizumab and ocrelizumab suggest potential benefits for EID in reducing treatment burden and associated risks, without sacrificing therapeutic efficacy. Despite limitations, these studies contribute to evolving MS treatment paradigms, indicating the importance of tailored dosing strategies to enhance patient care and outcomes. As the field develops, further studies in larger cohorts with longer follow-up times are needed to validate these preliminary findings, ensure longer-term efficacy, improve dosing guidelines and optimise care.

